# Ankylosing spondylitis and the gut microbiome: future research hotspots and trends

**DOI:** 10.3389/fimmu.2026.1784757

**Published:** 2026-05-05

**Authors:** Xingxiao Yin, Hao Peng, Yanqi Li, Yanping Song, Na Yao, Zhen Shen, Hongbo Chen, Li Huang, Pengcheng Li, Zhijuan He, Qigang Chen

**Affiliations:** 1 School of Physical Education, Yunnan Normal University, Kunming, China; 2 Department of Rehabilitation Medicine, The Third Affiliated Hospital of Yunnan University of Chinese Medicine (Kunming Municipal Hospital of Traditional Chinese Medicine), Kunming, China

**Keywords:** ankylosing spondylitis, bibliometrics, gut microbiota, inflammation, short-chain fatty acids

## Abstract

**Background:**

Ankylosing spondylitis (AS) is an autoimmune disease. Its exact cause remains unclear. It is generally believed to result from a combination of genetic and environmental factors, as well as immune disorders. However, growing evidence suggests that the gut microbiota plays a key role in the pathogenesis of AS. Therefore, this study aims to systematically analyze the current state of research on AS and the gut microbiome. It also explores future research hotspots.

**Methods:**

We searched the Web of Science Core Collection (WoSCC) and PubMed databases, including relevant literature on AS and the gut microbiome published up to January 1, 2026. We then performed a visualized bibliometric analysis using CiteSpace, VOSviewer, and Bibliometrix software.

**Results:**

The WoSCC dataset included 165 articles. Both the annual publication volume and citation counts showed an upward trend. Brown, Ma, and Liu B were the most productive authors. Regarding country output, China ranked first with 60 articles, followed by the USA with 36. Major contributing institutions were also primarily located in China and the USA. Current research hotspots focus on inflammation, Mendelian randomization, HLA-B27, probiotics, and short-chain fatty acids. A validation analysis using the PubMed database (115 articles) yielded results consistent with the WoSCC findings.

**Conclusion:**

Our study provides key insights into the relationship between the gut microbiota and AS. It clarifies current research hotspots and development trends. Future researchers should conduct prospective studies to confirm causality and combine multi-omics analysis to reveal underlying molecular mechanisms.

## 1 Introduction

Ankylosing Spondylitis (AS) is a chronic autoimmune disease marked by inflammation. It mainly affects the axial skeleton and is part of the spondyloarthritis spectrum (1, 2). Its main features are inflammatory back pain, enthesitis, and progressive damage to the sacroiliac joints and spine (3). Uncontrolled inflammation causes pathological fibrosis and ossification. This leads to the characteristic “bamboo spine” change (4). The resulting damage limits spinal mobility and function. It also harms the patient’s quality of life. AS mainly affects young adults (ages 20-30), with a clear male susceptibility (5–9). Human leukocyte antigen HLA-B27 is the main genetic marker. However, genetics alone does not fully explain how the disease begins and develops (10–13). Treatments range from non-steroidal anti-inflammatory drugs to biologics targeting TNF-α and IL-17. These can relieve symptoms, but stopping bone spur formation and achieving a cure remain challenging (14, 15). Identifying environmental triggers and new pathogenic mechanisms beyond genetics is crucial. This supports the development of precise intervention strategies.

Recently, advances in microbiome research have made the role of the gut microbiome in autoimmune diseases a research focus (16). Clinical evidence shows that most AS patients have subclinical gut inflammation. Also, AS and inflammatory bowel disease (IBD) share many genetic and immunological features. This strongly supports the “gut-joint axis” hypothesis (17–20). Regarding the mechanism, gut dysbiosis occurs. This appears as a decrease in beneficial bacteria that produce short-chain fatty acids (SCFAs) and an increase in pathogenic bacteria. This dysbiosis may damage the gut barrier and cause bacterial products to translocate. This then activates the IL-23/IL-17 signaling pathway and induces systemic inflammation (21–23). In addition, cross-reaction between specific bacterial antigens and HLA-B27 molecules is considered a potential trigger of autoimmune attacks (24). These findings suggest that gut microbiota is not merely a concurrent phenomenon of AS. It is likely a potential driver and a therapeutic target.

Research literature on AS and gut microecology is accumulating rapidly. However, the knowledge system in this field remains fragmented. Studies differ on changes in bacterial flora. The research focus is also shifting. It is moving from simple descriptions of floral abundance to multi-omics analysis and causal inference (such as Mendelian randomization). We need to clarify this evolution and identify research hotspots. Therefore, this study used CiteSpace, VOSviewer, and Bibliometrix. We systematically analyzed relevant literature in Web of Science Core Collection(WoSCC) and PubMed through January 2026. This study aims to build knowledge maps and show global cooperation, knowledge evolution, and frontier trends. We specifically aim to identify the key shift from “cross-sectional association” to “causal mechanism analysis.” This will offer a reference for future research and clinical translation.

## 2 Materials and methods

### 2.1 Database resources

We obtained literature for this study from the WoSCC and PubMed databases. The search period covered the time from the establishment of each database until January 1, 2026.

### 2.2 Search strategy

We used PubMed’s Medical Subject Headings (MeSH), a controlled vocabulary thesaurus, to gather literature. We followed the search rules of WoSCC and PubMed. Main keywords included “Ankylosing spondylitis,” “gut microbiome,” and related terms (see **Supplementary Materials 2** for details).

### 2.3 Literature screening and inclusion criteria

This study included only research articles and limited the language to English. After searching with the keywords, two researchers independently screened the results. We initially obtained 331 records from WoSCC and 248 from PubMed. We excluded articles unrelated to gut microbiome and AS, non-research article types (such as reviews, editorials, letters, and meeting abstracts), duplicate publications, and non-English literature. We exported data directly from public databases, so we did not require ethics committee approval. We finally selected 167 articles from WoSCC and 115 from PubMed. **Supplementary Figure 1** presents the detailed screening process.

### 2.4 Analysis methods

We selected 165 articles that met the inclusion criteria. All records were exported and downloaded in Plain Text, Tab-delimited, and BibTeX formats. Files were named “download_XXX.txt,” where “XXX” represents a specific identifier. For data analysis, we used CiteSpace (version 6.1.R6). We extracted and organized burst keywords. We generated keyword timeline maps based on word frequency and built co-occurrence maps. We used VOSviewer (version 1.6.18) to identify countries, institutions, journals, and keywords related to the articles. We generated co-occurrence network maps. We used the bibliometrics package in R Studio 4.2.3 to visualize countries and authors. Additionally, we used Microsoft Excel 2019 for data management and trend analysis of publication output and co-citation data (including annual output). The 115 articles from the PubMed database were used to verify the results from WoSCC.

## 3 Results

### 3.1 Publication output and journal distribution

**Figure 1:**
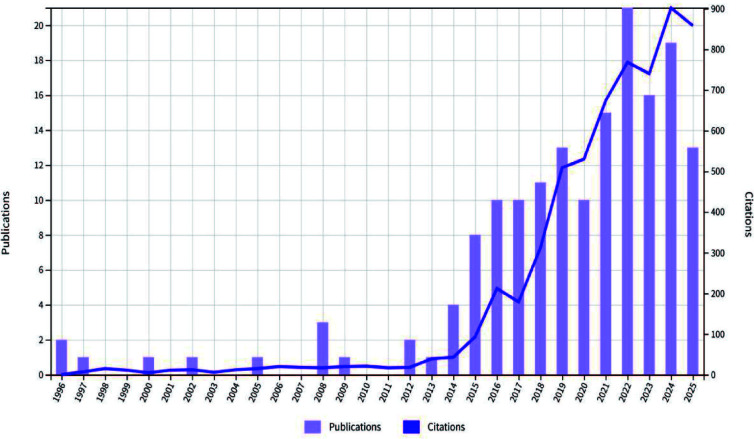
Annual publication and citation volume(WoSCC).

**Figure 2:**
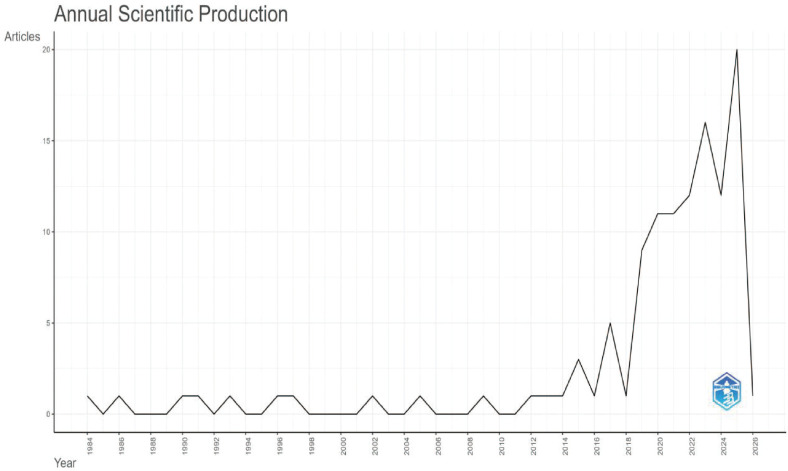
Annual publication and citation volume(Pubmed).

**Figure 3:**
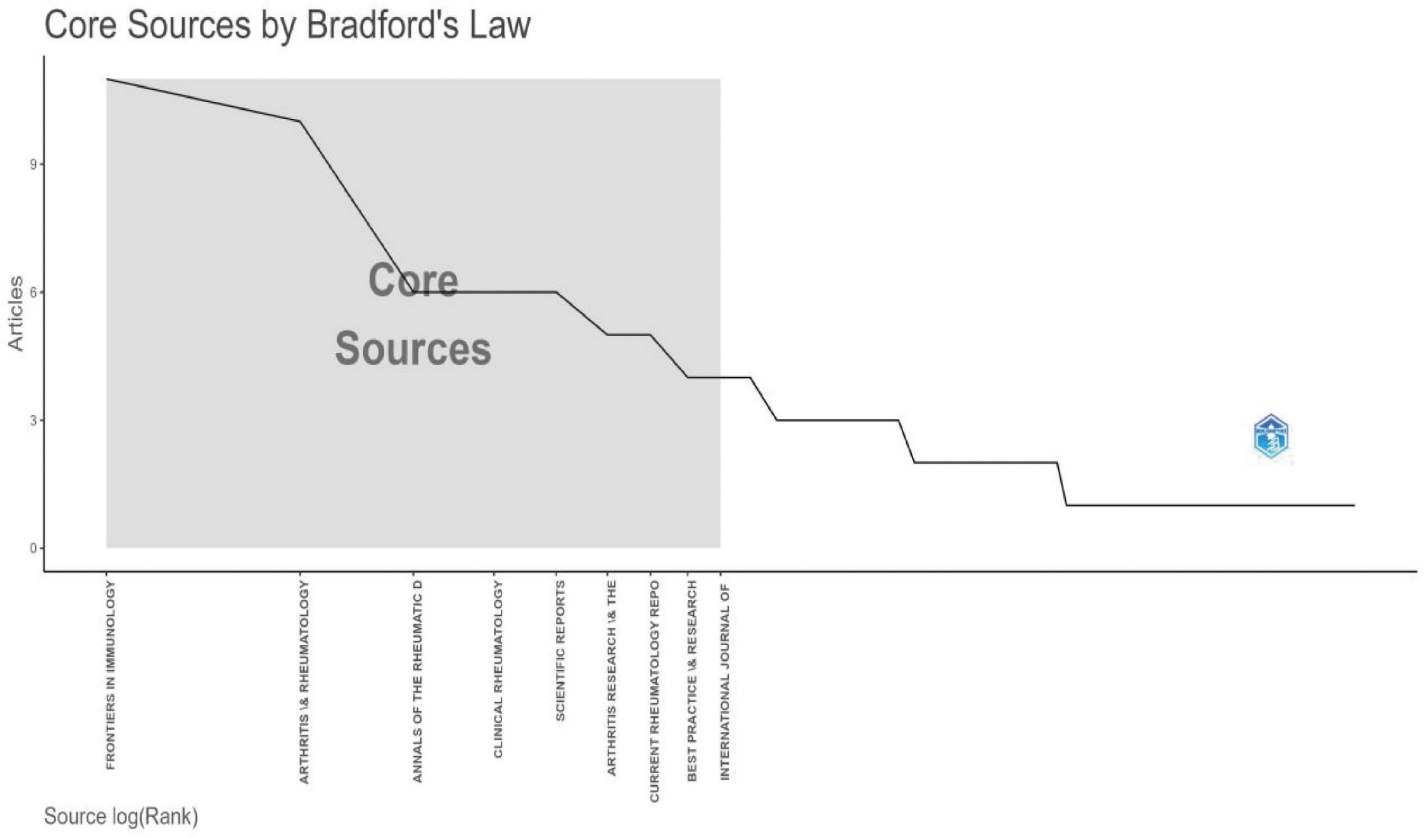
Core journals(WoSCC).

**Figure 4:**
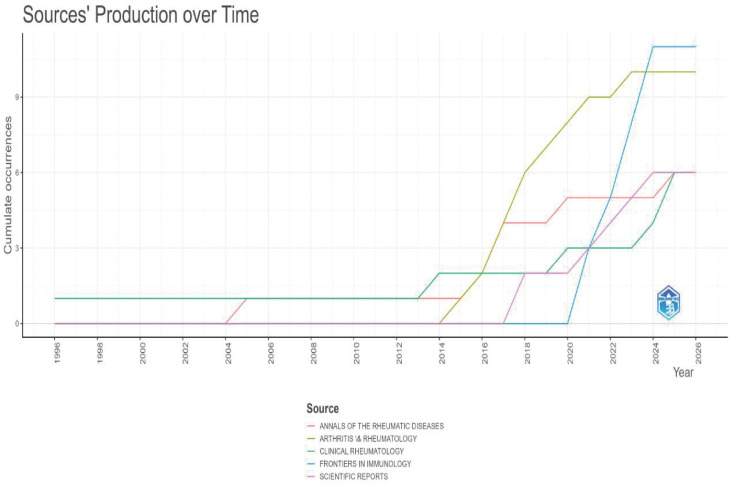
Number of publications in core journals(WoSCC).

**Figure 5:**
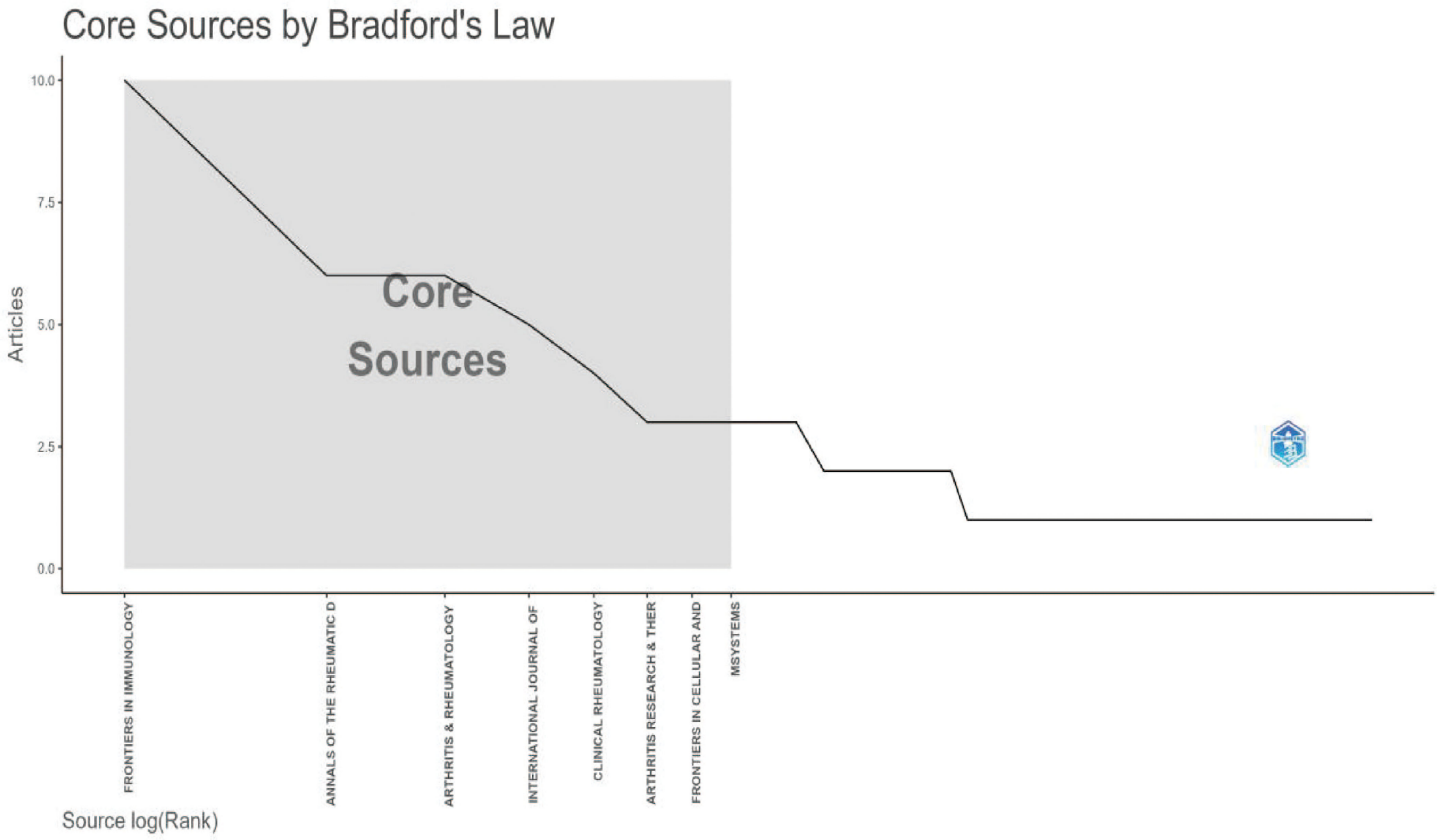
Core journals(Pubmed).

**Figure 6:**
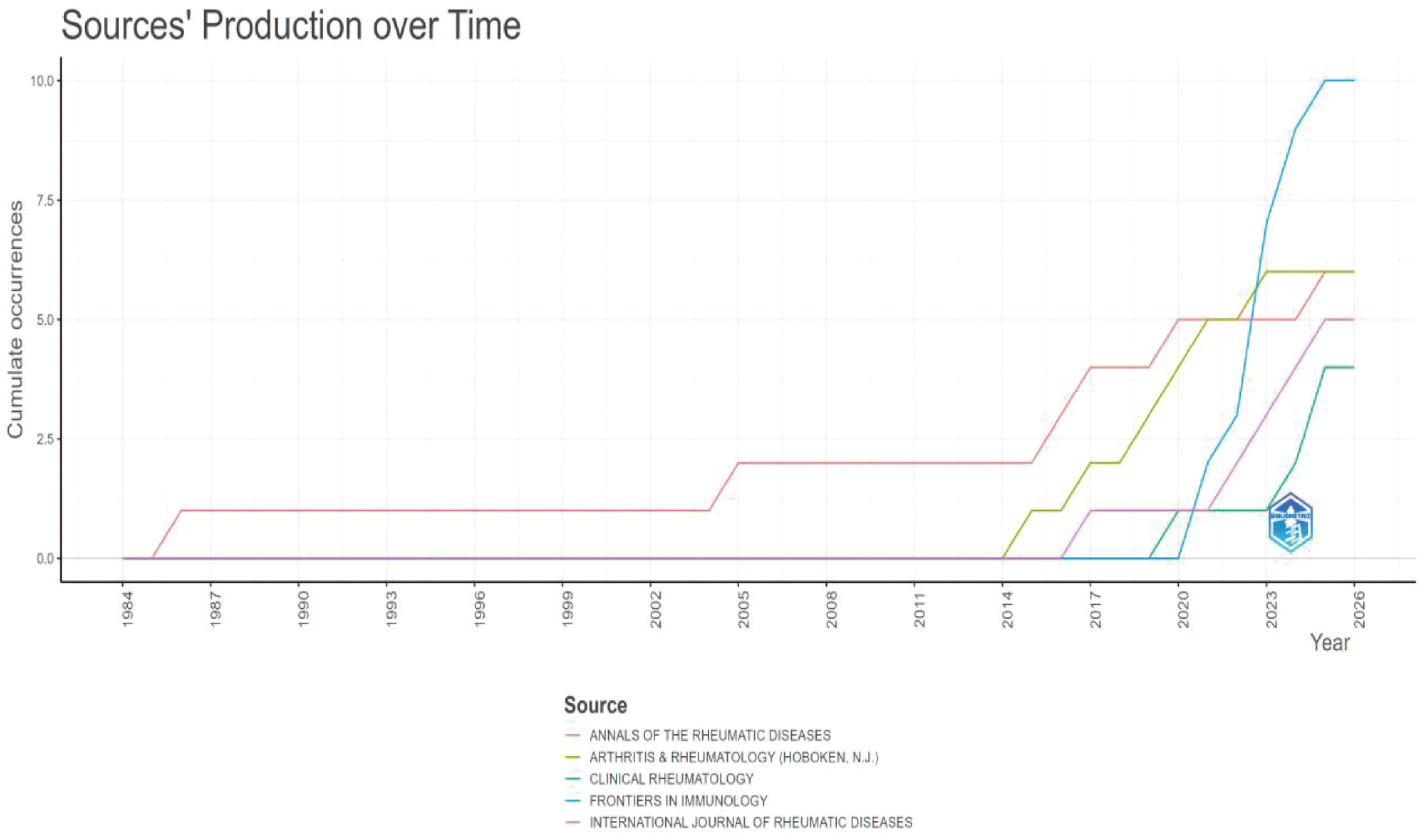
Number of publications in core journals(Pubmed).

This study included 165 publications. The first article in this field was published in 1996. It was titled “The Use of a Low Starch Diet in the Treatment of Patients Suffering from Ankylosing Spondylitis.” This paper discussed dietary intervention. It noted that a low-starch diet could reduce inflammation and symptoms in AS patients. Overall, both the volume of publications and citations in this field are rising (**Figure 1**). This trend matches the results in **Figure 2**. Research articles on the gut microbiome and AS were published in 87 journals (**Supplementary Figure 2**). Among the top ten journals (**Supplementary Table 1**), *Annals of the Rheumatic Diseases* has the highest impact factor (20.6). *Frontiers in Immunology* has the highest number of publications, with 11 articles. Furthermore, according to Bradford’s Law, specific journals form the core zone. Their publication volume is growing annually. This indicates that the field remains a research hotspot (**Figures 3**, **4**). The analysis results of **Figures 5**, **6** further confirm the findings of **Figures 3**, **4**.

### 3.2 Author visualization analysis

**Figure 7:**
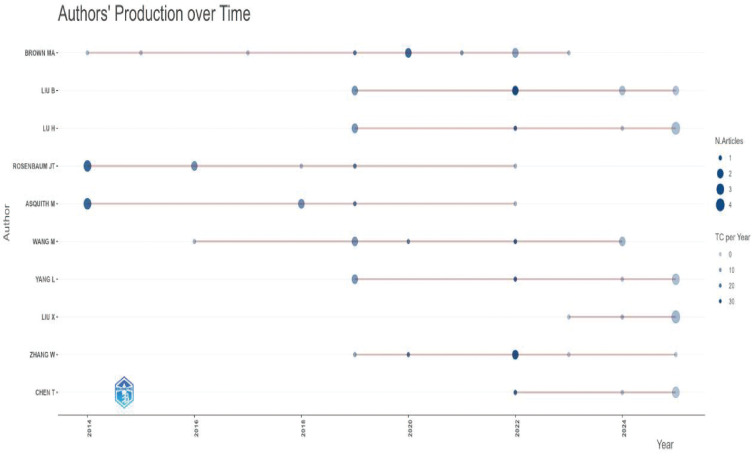
Number of publications by the top ten authors(WoSCC).

**Figure 8:**
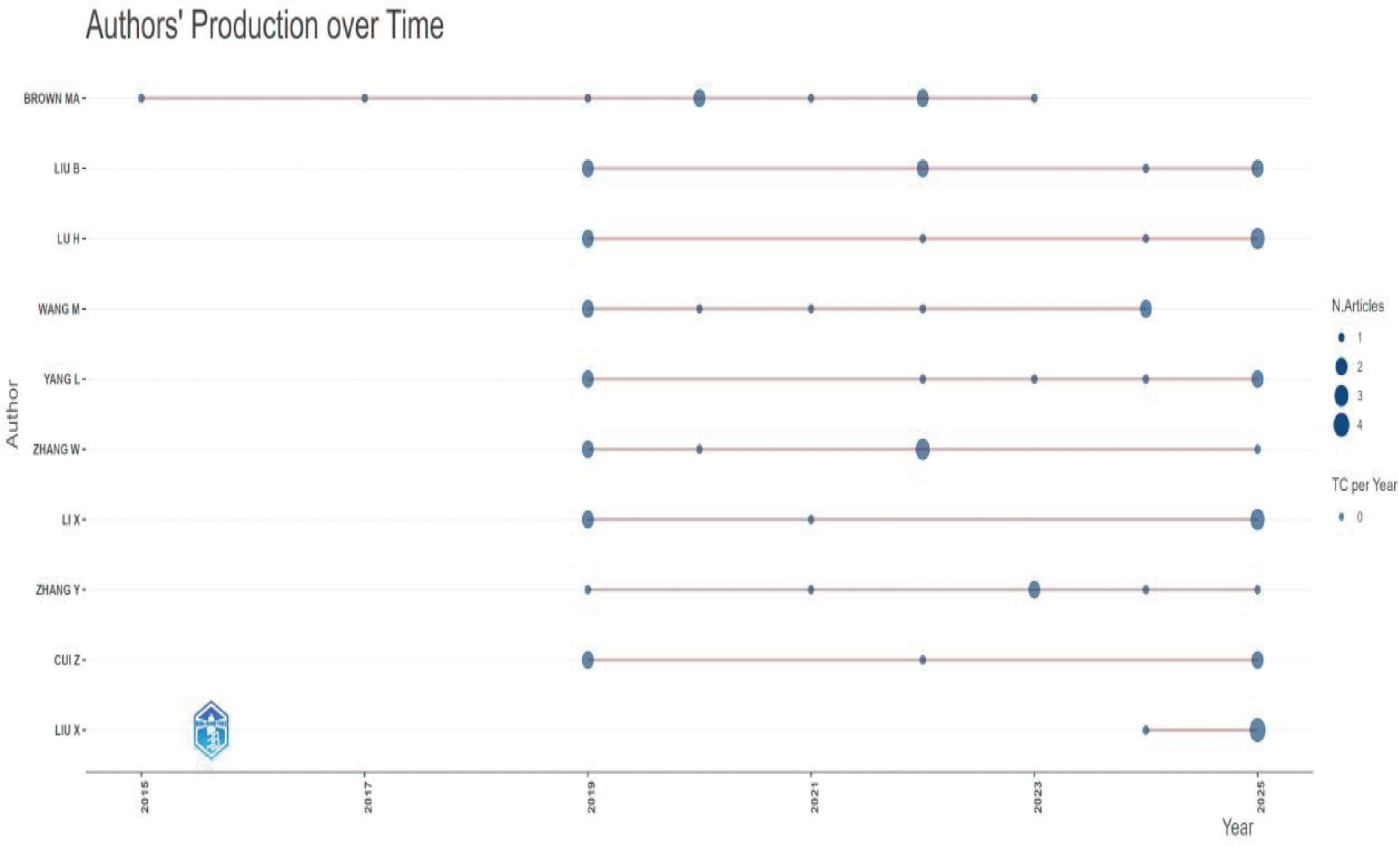
Number of publications by the top ten authors(Pubmed).

A total of 1,077 authors contributed to articles in this field (see **Supplementary Figure 3**). We identified the top ten authors by publication volume (as shown in **Figures 7**, **8**). Brown, M.A., and Liu, B. lead the list with the most publications.

### 3.3 Country (or region) and institutional cooperation analysis

**Figure 9:**
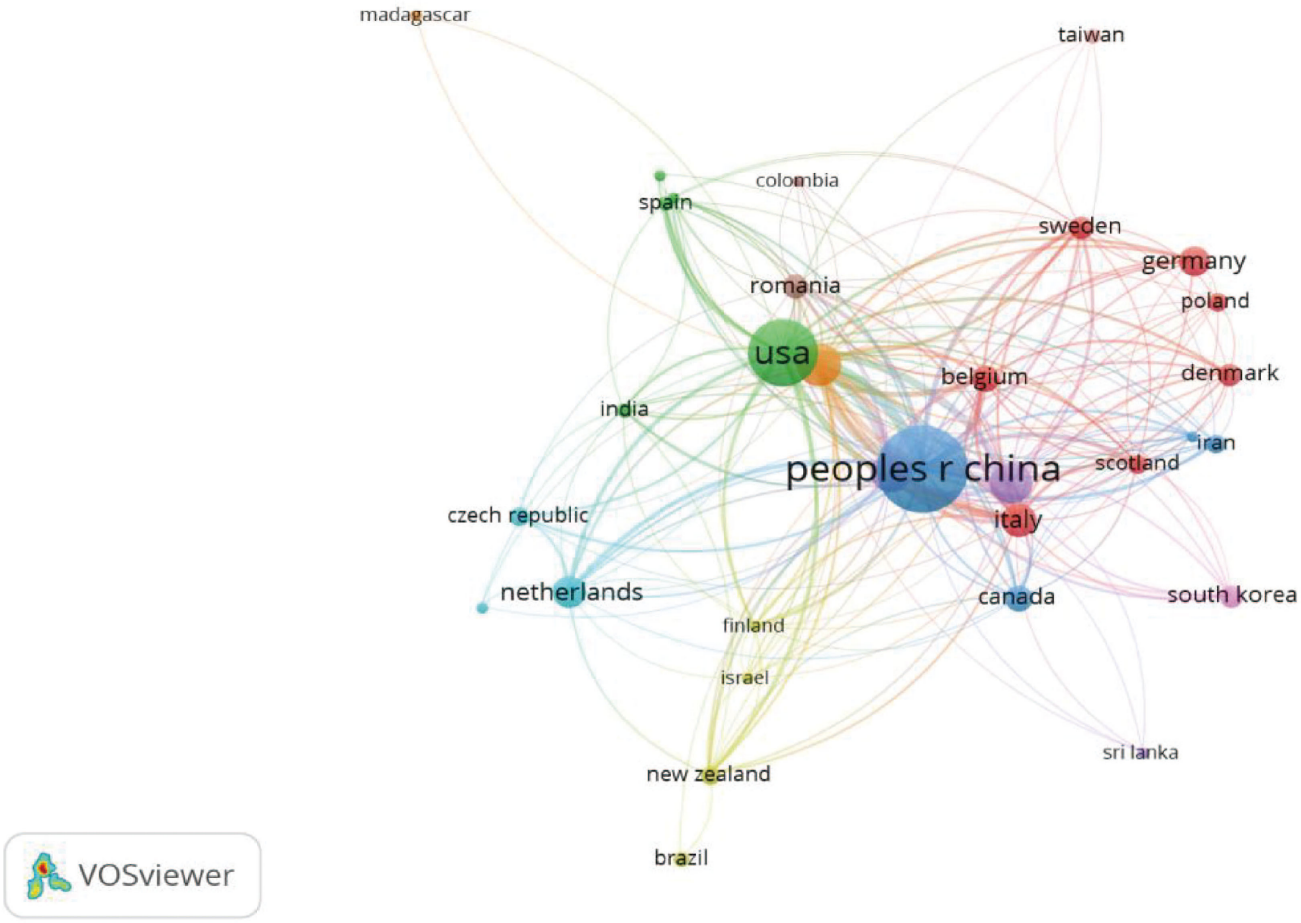
Visualization map of countries (node size represents the number of articles issued by countries, connection lines represent the degree of Cooperation,WoSCC).

**Figure 10:**
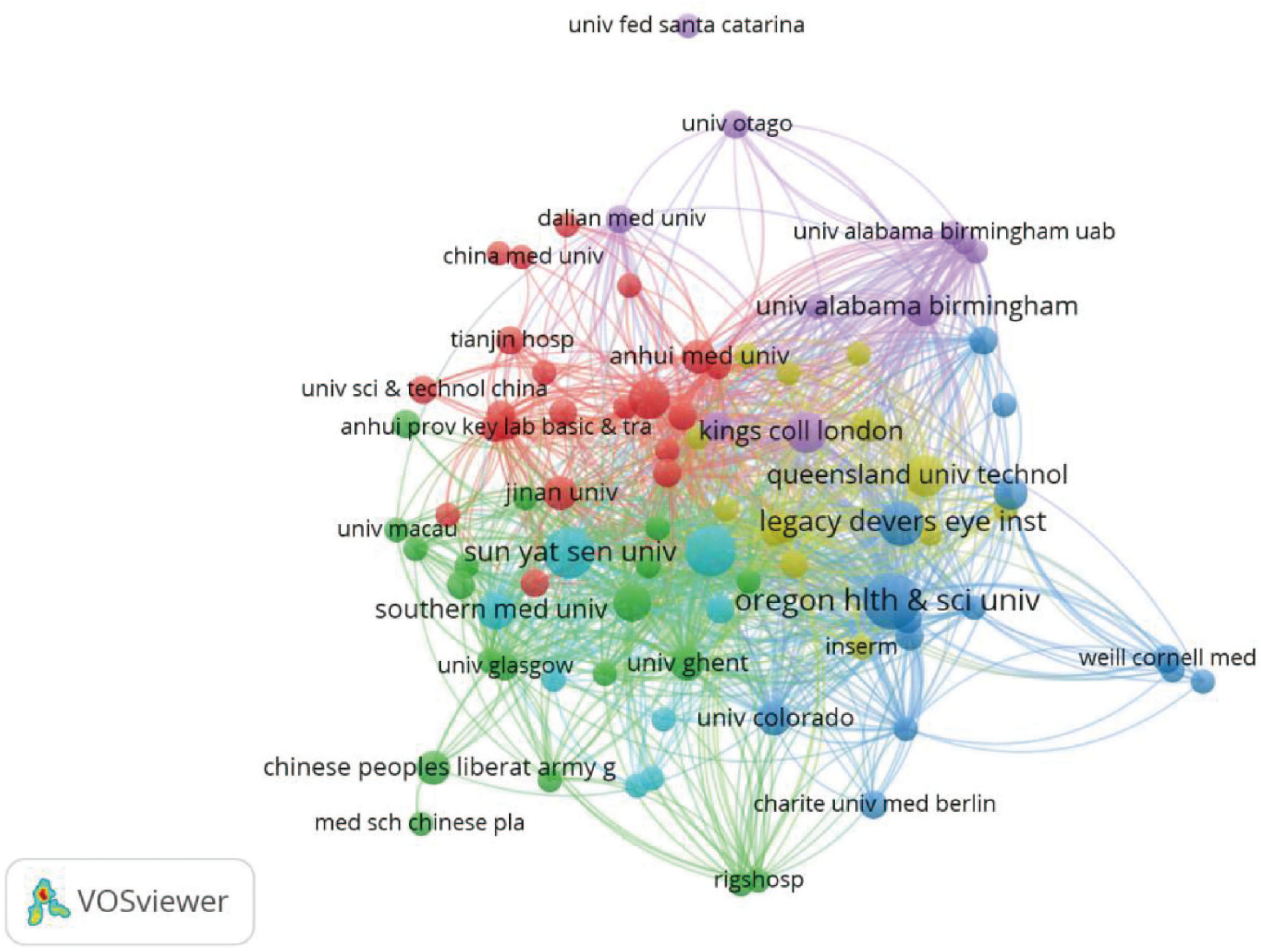
Visualization map of institutions (node size represents the number of articles issued by institutions, and lines represent the degree of collaboration,WoSCC).

As shown in **Figure 9**, researchers from 35 countries (or regions) contributed to research on AS and the gut microbiome. China leads with 60 publications. The United States follows with 36, and the United Kingdom with 15. International cooperation is mainly concentrated among these high-output countries. As shown in **Figure 10**, 374 institutions published relevant literature. The main research institutions are in China and the United States.

### 3.4 Literature citation analysis

We analyzed the top 10 most cited articles (see **Supplementary Table 2**). Two studies used genome sequencing technology (e.g., “Quantitative metagenomics reveals unique gut microbiome biomarkers in ankylosing spondylitis,” “Metagenomic profiling of the pro-inflammatory gut microbiota in ankylosing spondylitis”). Six studies used 16S rRNA gene sequencing and analysis. These involved papers, such as “Dysbiosis of the gut microbiome in ankylosing spondylitis,” “Fecal microbiota study reveals specific dysbiosis in spondyloarthritis,” and others, focus on IgA-coated *E. coli*, *Dialister*, and HLA-B27 effects in rats and humans. One study used 16S rDNA gene sequencing (“Microbiota changes associated with abnormal humoral immune response to commensal organisms in enthesitis-related arthritis”). Another used molecular biology detection methods (“Dysbiosis and zonulin upregulation alter gut epithelial and vascular barriers in patients with ankylosing spondylitis”). These studies found that the abundance of *Prevotella melaninogenica*, *Prevotella copri*, and *Prevotella* sp. significantly increased in AS patients. In addition, studies showed significant differences in the microbial community of the terminal ileum between AS patients and healthy controls. This was mainly due to increases in five families (*Lachnospiraceae*, *Ruminococcaceae*, *Rikenellaceae*, *Porphyromonadaceae*, and *Bacteroidaceae*) and decreases in two families (*Veillonellaceae* and *Prevotellaceae*). In patients with spondyloarthritis, the abundance of *Ruminococcus gnavus* increased two to three times. Furthermore, adhesive and invasive bacteria were observed in the gut of AS patients. The bacterial score was significantly correlated with gut inflammation. However, few studies currently use metabolomics, proteomics, or transcriptomics. There is also little in-depth discussion on the role of fungi and viruses in AS.

### 3.5 Keyword analysis

**Figure 11:**
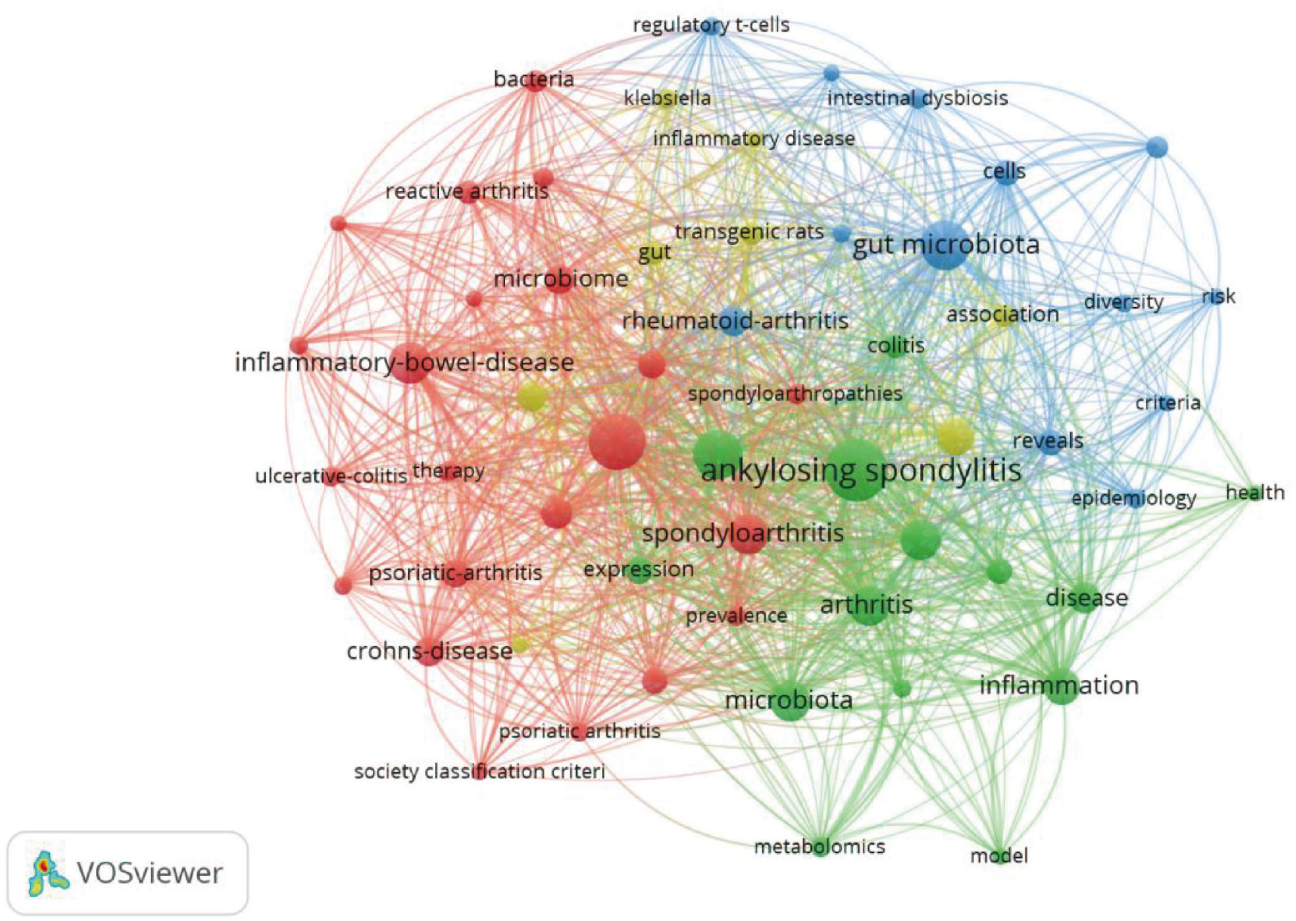
Keyword visualization graph (node size represents the frequency of keyword occurrence, and lines represent the degree of cooperation,WoSCC).

**Figure 12:**
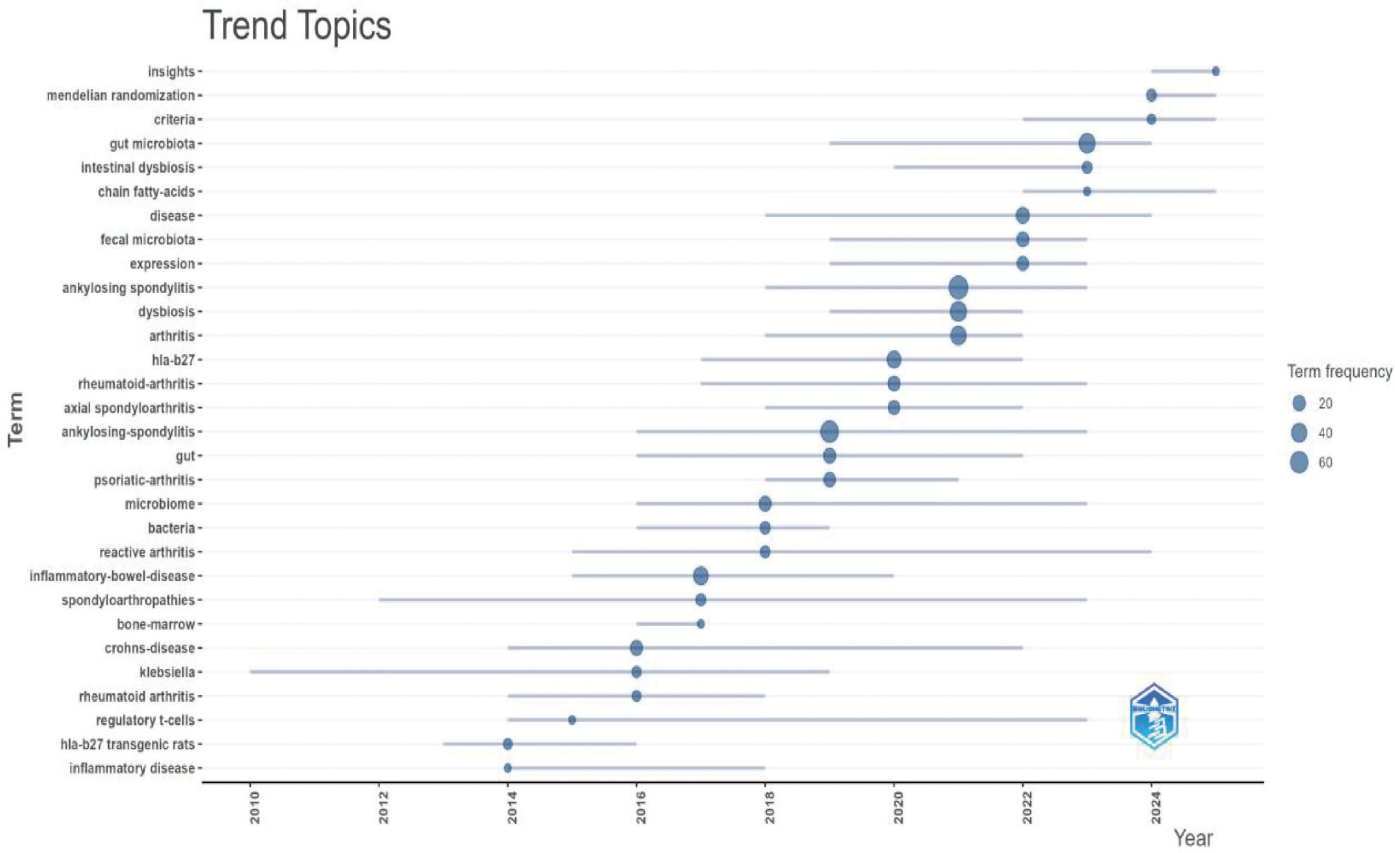
Keyword outbreak words(WOSCC). When the end is highlighted in blue, it signifies that the institution will be a prominent publishing organization in the future.

**Figure 13:**
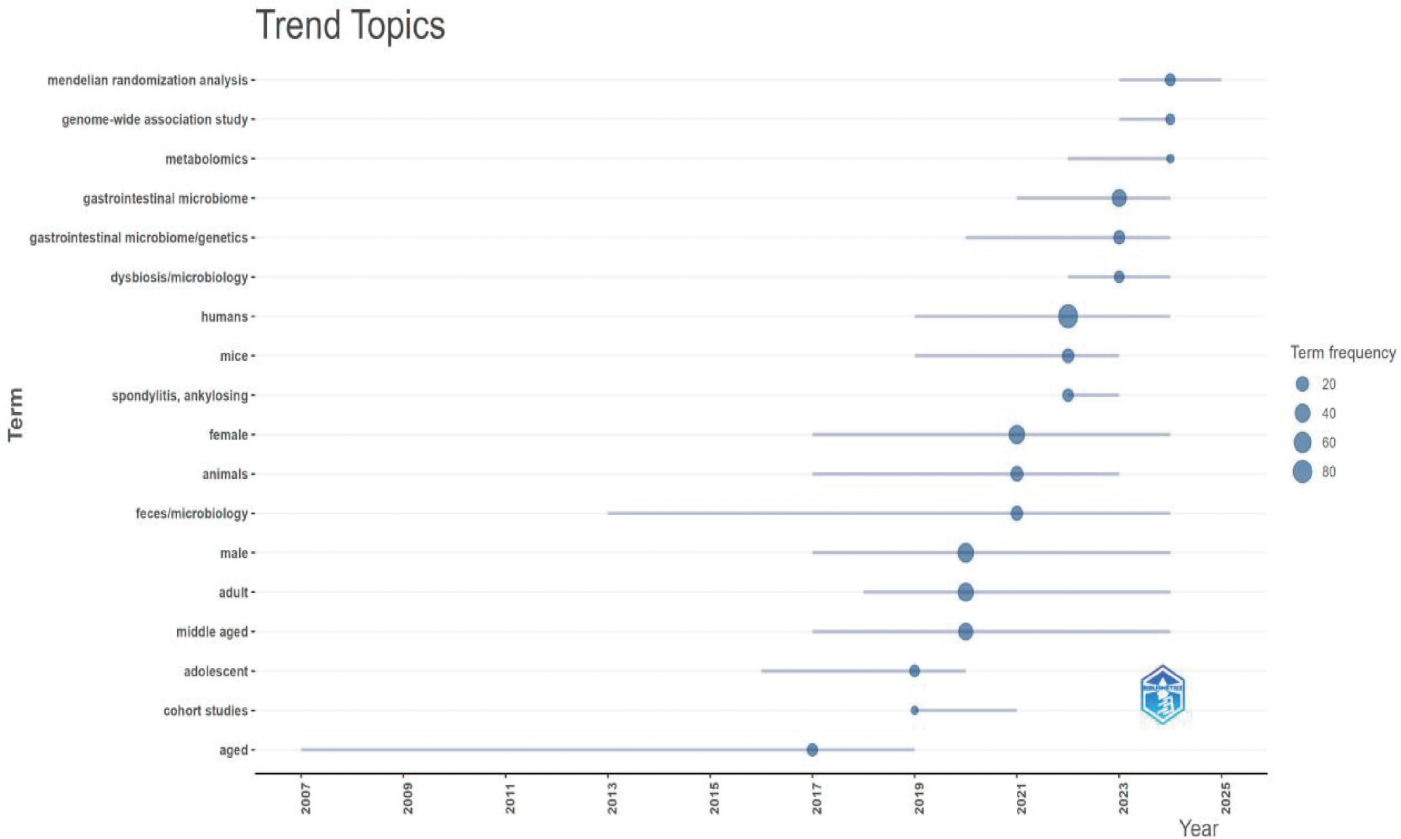
Keyword outbreak words(Pubmed). When the end is highlighted in blue, it signifies that the institution will be a prominent publishing organization in the future.

**Figure 14:**
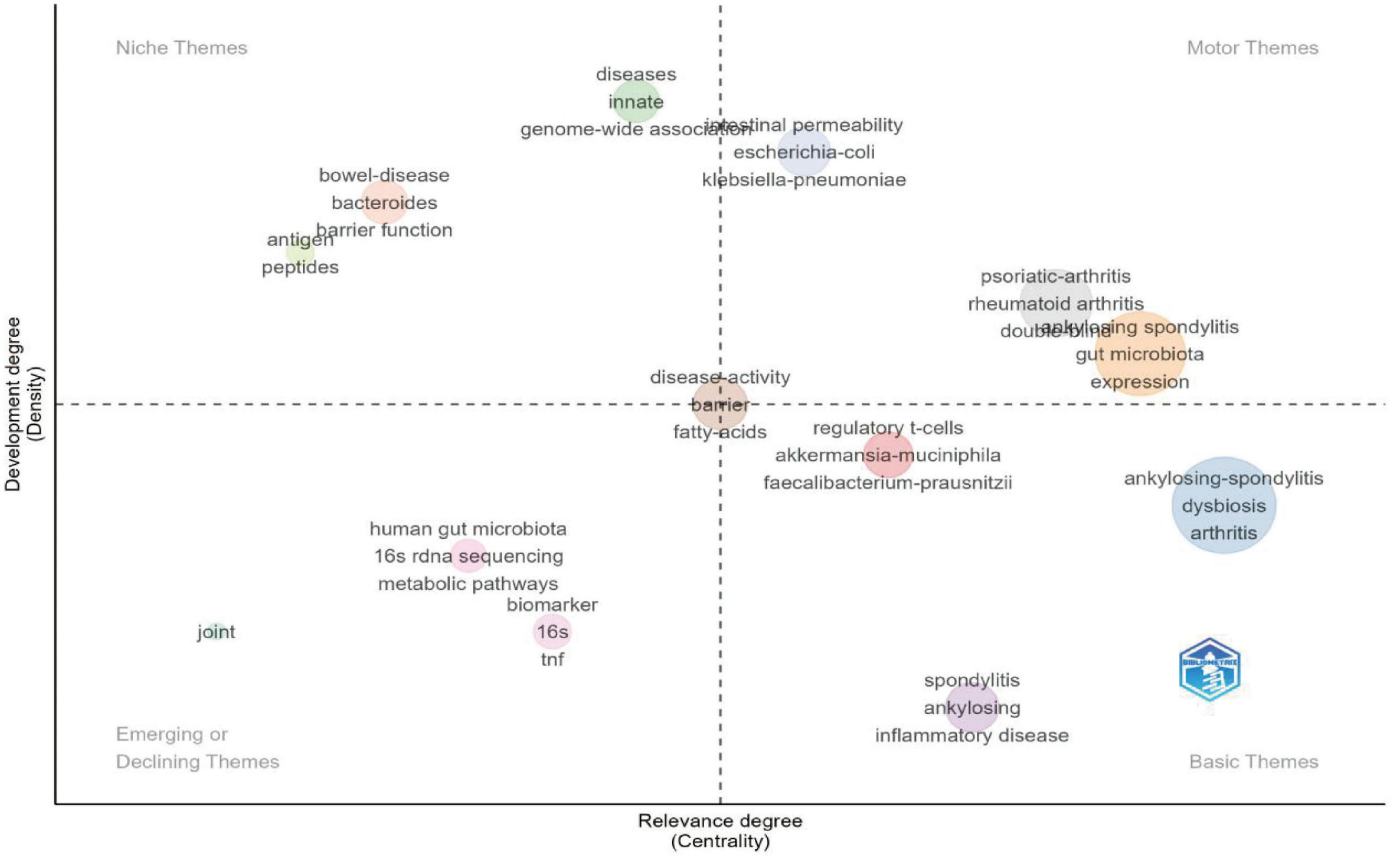
Theoretical map outlining the research themes in Thematic Map of Research Themes on Ankylosing Spondylitis and the Gut Microbiome in WoSCC and gut microbiome(WoSCC).

This study extracted 729 keywords (**Figure 11**). Besides the main subject terms, “inflammation,” “HLA-B27,” “pathogenesis,” and “inflammatory bowel disease” were the most frequently used. Burst keyword analysis revealed that “MR” is a potential future research hotspot (**Figures 12**, **13**; **Supplementary Figure 4**). We used the thematic map module (**Figure 14**) to explore current themes and future directions. We found the following patterns: The first quadrant (mature core themes) is dominated by “gut microbiome” and “ankylosing spondylitis.” The second quadrant contains widely researched themes with weak links to the core network, such as “disease” and “bowel disease.” The third quadrant gathers themes with low heat and low relevance (such as “16S rDNA sequencing”). The fourth quadrant highlights themes that are closely linked but currently have lower heat. These may become future hotspots, especially “inflammation,” “HLA-B27,” and “inflammatory bowel disease”.

## 4 Discussion

### 4.1 General information

In the past 20 years, the role of the gut microbiome in autoimmune diseases has gained attention. This is especially true for AS pathology. This study used CiteSpace, VOSviewer, and Bibliometrix tools. We performed a dual-database search and de-duplication of literature in WoSCC and PubMed up to January 1, 2026. We finally included 165 core articles. We systematically reviewed the publication trends, geographic distribution, cooperation networks, and evolution of research hotspots in this field.

From a timeline view, research in this field has developed in stages. As early as 1996, scholars began exploring the effects of a low-starch diet on relieving inflammatory symptoms in AS. This marked the start of relevant research (25). Subsequently, with the spread of 16S rRNA sequencing and metagenomic technology, the volume of literature accumulated steadily. Especially after 2021, the annual volume of publications and citations showed a significant upward trend. This trend reflects the growing academic attention to the potential role of the “gut-joint axis” in AS pathogenesis as high-throughput sequencing technology matures.

Regarding geographic distribution and institutional cooperation, China and the United States are the main contributors. Bibliometric results show that China leads with 60 publications, followed closely by the US with 36. Major contributing institutions, such as Sun Yat-sen University and Oregon Health & Science University, have formed a close academic cooperation network between the two countries. This distribution indicates that research teams in China and the US are leading the field’s development.

Regarding publication channels and core authors, the included literature is distributed across 87 journals. *Frontiers in Immunology* published the most articles. *Annals of the Rheumatic Diseases*, a top rheumatology journal, also published many high-impact studies. Scholars like Brown, MA, and Liu B remain highly active in this field. Analysis of highly cited literature reveals that existing research mainly uses sequencing technology. These studies reveal specific changes in the gut flora of AS patients. For example, there is an increase in *Prevotella* abundance and a decrease in SCFA-producing bacteria, such as *Lachnospiraceae*. This dysbiosis is closely associated with impaired gut barrier function and systemic inflammation (26).

Keyword co-occurrence and burst analysis further reveal a shift in research focus. Besides classic themes like “inflammation,” “HLA-B27,” and “pathogenesis,” “MR” is a buzz term in recent years. This indicates a change in research mode. It is moving from early, simple cross-sectional correlation-based descriptions to the use of genetic tools to explore the causal link between gut flora and AS.

Although progress has been made in identifying pathogenic bacteria and correlation analysis, limitations exist. The integrated application of multi-omics (such as metabolomics and transcriptomics) is relatively rare. There is also insufficient attention to non-bacterial microbiomes, such as fungi and viruses. Future research should focus more on mechanism exploration. It should combine multi-omics technology to clarify the specific molecular pathways by which bacterial metabolites (such as SCFAs) regulate host immunity. Researchers should also use methods like MR to verify causality. This will provide a reliable scientific basis for the precise treatment of AS based on gut microecology.

### 4.2 Research hotspots

#### 4.2.1 Inflammation

AS is a chronic inflammatory disease related to genetics, immunity, and microbial factors. Impaired gut barrier function leads to the migration of bacteria and their products. This activates a systemic inflammatory response. Specifically, it acts through the inflammasome pathway, promoting the secretion of IL-1β and IL-18. This subsequently affects the IL-23/IL-17 axis (27). The IL-23/IL-17 axis and related cytokines are considered linked to the etiology of Spondyloarthritis (28). This not only triggers local gut inflammation but is also associated with the systemic inflammatory state in AS patients. For example, under stimulation by bacterial products, innate lymphoid cells (ILCs), especially ILC3, can produce IL-17 to trigger an inflammatory response. This links the gut microbiome with local/systemic immunity (29). IL-23 can induce Th17 cells to produce IL-17, and Paneth cells are an important source of IL-23 (30). Paneth cells produce IL-23 and IL-17 after recognizing an altered microbiome. IL-23 promotes the differentiation of Th17 and ILC3 cells. These activated cells promote elevated IL-17 levels, thereby maintaining inflammation (29). Therefore, in addition to traditional non-steroidal anti-inflammatory drugs and TNF-α inhibitors, treatments such as gut microbiome regulators and gut barrier repair agents show promise. These new treatments may bring hope, especially for patients who do not respond well to traditional therapies.

#### 4.2.2 Probiotics and short-chain fatty acids

Given the important role of the gut microbiome in AS pathogenesis, regulating gut microbiota is a promising therapeutic approach. Zhang HT et al. found that the microbiome of AS monkeys showed increased abundance of *Clostridia*, *Clostridiales*, *Ruminococcaceae*, and *Prevotella 2* (31). The increase in *Prevotella 2* leads to reduced SCFA production (especially butyrate). This causes decreased expression of tight junction proteins and increased gut permeability. The formation of “leaky gut” allows bacterial lipopolysaccharides to enter the circulation. This promotes the release of inflammatory factors and drives AS progression. Probiotics have been proven to improve the microbiome by altering the gut environment, inhibiting the growth of harmful bacteria, and preventing further immune system damage caused by inflammatory diseases (32). However, many issues remain regarding the application of probiotics in AS. First, sample sizes are small, and quality is low. We need more multi-center, large-sample clinical control trials. Additionally, we need to consider factors such as disease stage, diet, and ethnicity. Finally, the anti-inflammatory effect of probiotics relies heavily on dosage and strain. Different strains may have different functions with the host, and the duration of intervention matters. Furthermore, metabolites of beneficial bacteria, such as SCFAs (especially butyrate), have significant anti-inflammatory and immune-modulating effects. SCFAs are fatty acids with fewer than 5 carbon atoms. Acetate, propionate, and butyrate are most abundant in the gut environment (33). Butyrate can upregulate tight junction protein expression to repair the gut barrier and reduce gut leakage. It can also directly inhibit AS inflammation through several pathways. Regulating immune cells inhibits the macrophage TLR4/NF-κB pathway and reduces IL-6 and TNF-α release. It promotes Treg cell differentiation in the gut mucosa and secretes IL-10 to block activation of the IL-23/IL-17 axis. Through epigenetic regulation, it inhibits histone deacetylase (HDAC) and downregulates the transcription of pro-inflammatory genes. This inhibits the inflammatory response at the molecular level (34). Therefore, we can explore AS treatments from the perspective of probiotics and SCFAs. These are future research hotspots for AS and the gut microbiome.

#### 4.2.3 HLA-B27

HLA-B27 is the core genetic susceptibility factor for AS. Recent studies have gradually revealed its coordinated regulatory relationship with gut microecology, the gut barrier, and inflammatory pathways. HLA-B27-positive individuals (including healthy carriers) already have a characteristic imbalance in gut flora composition. This imbalance appears before the onset of AS (35). In addition, some microbial components in the gut flora of AS individuals are very similar to AS self-epitopes (36). When the gut barrier is damaged, these bacterial antigens enter the circulation. HLA-B27 molecules can present them to immune cells, triggering a cross-reactive immune response and advancing AS progression. Therefore, future research can investigate the causal relationship between “genetic background (HLA-B27) - gut microbiome - inflammatory response.” This will provide a theoretical basis for early screening of AS (e.g., combining HLA-B27 with flora markers) and for precise intervention.

### 4.3 Research trends: deep transformation from association description to functional mechanism investigation

#### 4.3.1 Mendelian randomization and causal inference

The keyword burst analysis in this study shows that “MR” is a significant growth point in recent AS research. This trend reflects that the current academic focus is shifting. It is moving from traditional cross-sectional observational studies to using genetic variations to infer the causal relationship between gut flora and AS. Traditional studies are easily disturbed by confounding factors (such as diet and medication). In contrast, MR uses genetic instrumental variables. This more strongly supports the idea that gut dysbiosis may be a driver of AS onset rather than just a result of the disease.

At the specific flora level, several MR studies have used genetic approaches to screen for potential pathogenic and protective bacteria associated with AS (37–40). For example, studies found that the genetically predicted abundance of *Actinobacteria* and specific subgroups of *Ruminococcaceae* (such as *Ruminococcaceae NK4A214 group*) is positively correlated with AS risk (38, 39). This matches clinical observations. It suggests that these bacteria may promote the release of pro-inflammatory factors by activating the NF-κB signaling pathway, thereby contributing to disease development (41, 42).

Conversely, bacteria such as *Lactobacillaceae* and *Rikenellaceae* have potential protective effects (40). This protective effect may be closely related to their metabolic functions, especially the ability to produce SCFAs (43). As mentioned earlier (Section 4.2.2), SCFAs (especially butyrate) can maintain gut barrier integrity and regulate immune responses (44). MR study evidence shows that reduced genetic susceptibility in these SCFA-producing bacteria leads to a weakened host anti-inflammatory response. This further supports the view that “insufficiency of bacterial metabolites is a risk factor for AS” (45).

Additionally, MR analysis provides a genetic explanation for the close link between AS and IBD. Clinical data show that many AS patients have subclinical gut inflammation (46). Research by Ding et al. used multi-cohort MR analysis. After removing horizontal pleiotropy, they confirmed at the genetic level that IBD is an independent risk factor for AS (47). This validates the “gut-joint axis” hypothesis. In genetically susceptible individuals, genetic defects in gut barrier function may cause abnormal activation of gut immune cells. These cells may then migrate to the joints via the bloodstream, triggering cross-reactive immune responses against shared antigens (48).

In summary, the rise of MR-related research not only verifies the causal role of gut flora in AS pathogenesis but also, from a genetic perspective, reveals that AS is a systemic inflammatory disease. These findings suggest that interventions targeting gut microecology (such as supplementing specific protective strains or metabolites) may not only relieve symptoms but also be a potential strategy to block disease progression.

#### 4.3.2 Metagenomics and functional analysis

The bibliometric analysis in this study shows that “Metagenomics” and related omics technologies have become research hotspots in recent years. This trend indicates that the research focus on AS gut flora is shifting from simple “flora abundance description” to “functional mechanism analysis.” Compared to traditional 16S rRNA sequencing, metagenomics can cover unculturable microbes. More importantly, gene functional annotation reveals the complex interaction between bacterial metabolites and the host immune system.

Functional research using metagenomics has identified significant changes in the metabolic characteristics of gut flora in AS patients. Specifically, there are abnormalities in energy metabolic pathways in the patient’s gut. This is shown as the enhanced tricarboxylic acid (TCA) cycle and oxidative phosphorylation, while glycolysis is weakened. This metabolic state may lead to increased local oxidative stress, favoring the overgrowth of facultative anaerobes (36). In addition, some studies observed active pathways of lipopolysaccharide (LPS) synthesis. As a typical endotoxin, LPS can directly induce joint inflammation (49). At the same time, levels of enzymes related to Vitamin B6 synthesis (such as pyridoxal 5’-phosphate synthase) decrease. This may weaken its ability to maintain the gut barrier, further increasing the body’s susceptibility to inflammation (50).

At the molecular mechanism level, metagenomic data further support the “molecular mimicry” hypothesis. This echoes the finding that “HLA-B27” is a core keyword in this study (51). Research shows that specific peptide segments of some gut commensal bacteria (such as *Prevotella copri* and *Klebsiella pneumoniae*) are highly similar to the host HLA-B27 molecular structure (52). In genetically susceptible individuals, these bacterial antigens may be misrecognized, triggering cross-reactive immune responses and initiating chronic inflammation (53).

Changes in the function of the flora finally converge on an immune regulation imbalance. This study shows that “inflammation” is one of the most frequent keywords and is closely associated with the IL-23/IL-17 axis. Enrichment of pro-inflammatory bacteria, such as Clostridium, can activate pattern recognition receptors (such as TLRs) and promote the release of pro-inflammatory factors, such as TNF-α and IL-17 (51). Meanwhile, the abundance of anti-inflammatory SCFA-producing bacteria (such as *F. prausnitzii*) decreases. This leads to weakened immune regulation ability. This “pro-inflammatory/anti-inflammatory” imbalance is considered a key factor driving systemic inflammation in AS (54).

Future research should pay more attention to the impact of population heterogeneity on the “microbiome-metabolism-immune” network. Given differences in HLA-B27 subtypes and ERAP1 gene loci across races, specific microecological evolution may be population-specific (55). Furthermore, the remodeling effect of clinical treatments (such as biological agents) on the function of the flora is also an important translational research direction. Finally, based on the integrated analysis of multi-omics data, we hope to shift from broad-spectrum anti-inflammatory treatment to personalized microecological intervention (such as precise diet and microbiota transplantation). This will provide new strategies for the whole-course management of AS.

### 4.4 Advantages and limitations

This study is the first bibliometric analysis focusing on the field of AS and the gut microbiome. By systematically reviewing literature from the past 20 years, we visually demonstrated the current state and evolution trends of this field. The main advantage of this study lies in the dual-database search strategy using WoSCC and PubMed. WoSCC provides authoritative citation data, facilitating co-citation analysis to identify core literature. PubMed, an authoritative database in biomedicine, covers a broader range of basic and clinical research. Compared to single-database studies, this complementary strategy can more effectively cover key literature in the field. This improves the reliability of research conclusions and helps researchers accurately grasp the trend from “flora description” to “mechanism exploration”.

However, this study still has certain limitations. First, although we used two major databases, we did not include Embase, Scopus, or Chinese databases (such as CNKI). Also, due to the restriction to English literature, we may have missed some local studies from non-English-speaking countries. Given that diet structure and ethnic differences are important factors influencing gut flora, missing data from these regions may compromise the completeness of the global perspective. Second, this study primarily relies on quantitative indicators such as publication volume, citation frequency, and keyword co-occurrence. This method reflects research popularity well, but cannot directly assess the experimental quality or clinical translational value of the articles. Highly cited literature attracts academic attention but is not necessarily of the highest quality of evidence. Third, although we manually screened to exclude irrelevant literature, AS often co-exists with diseases like IBD. Since both overlap in immune mechanisms, some keywords (such as “inflammation,” “gut dysbiosis”) are difficult to fully distinguish as targeting AS itself or its comorbid state. This may bring slight interference to keyword cluster analysis. Fourth, citation analysis typically lags. It takes time for a paper to gain many citations after publication. The data for this study are up to January 2026. This means high-quality recent results published between 2024 and 2025 (such as the latest microecological intervention clinical trials) may not be highlighted in the co-citation network due to insufficient accumulated citations. In summary, despite these limitations, the general trends and hotspot distribution revealed by this study are representative. They can provide valuable references for subsequent mechanism research and clinical translation.

## 5 Conclusion

This study systematically analyzed the academic landscape of AS and gut microecology for the first time by integrating WoSCC and PubMed databases. Bibliometric data show that research output in this field has grown exponentially over the past twenty years. It entered an explosion period, especially after 2021. China and the United States are major contributors, dominating the global scientific research cooperation network. Our analysis reveals a significant shift in research focus. It has deepened from early descriptions of single-flora abundance to discussions of the mechanisms of interaction among “inflammation,” “HLA-B27,” and microecology. In particular, the emergence of new hotspots such as “MR,” “short-chain fatty acids,” and “probiotics” marks a shift in the research paradigm. It is moving from cross-sectional correlation observation to causal inference and functional intervention. Although the theoretical framework of the “gut-joint axis” is maturing, current evidence is mostly limited to cross-sectional studies. Future research should prioritize prospective longitudinal cohort studies. This will help clarify the temporal causal relationship between gut dysbiosis and AS onset. At the same time, combining multi-omics technologies, such as metabolomics, to deeply analyze the specific molecular mechanisms by which flora metabolites regulate host immunity will be key. This is essential for translating basic research into precise microecological treatment.

## Data Availability

Publicly available datasets were analyzed in this study. This data can be found here: http://www.ncbi.nlm.nih.gov/PubMed/.
