# Supplementary material for: Ankylosing spondylitis and the gut microbiome: future research hotspots and trends
Source: Front Immunol. 2026 May 5;17:1784757. doi: 10.3389/fimmu.2026.1784757 (PMC13183638; doi:10.3389/fimmu.2026.1784757)
Supplement: Supplementary file 1 [file Table1.docx]

**Ankylosing spondylitis and the gut microbiome: Future research hotspots and trends.**

**Appendix 2**

**supplementary figure 1** **Retrieval process flowchart for the research** 2

**supplementary figure 2 Network visualization map of journal co-citation analysis generated by VOSviewer**. 3

**supplementary figure 3 The author co citation analysis network visualization generated by VOSviewer.** 4

**supplementary figure 4 Keywords outbreak** ５

**supplementary Table 1 Top 10 most productive journals.** ６

**supplementary Table 2 Top 10 highly cited literature**..............**７**


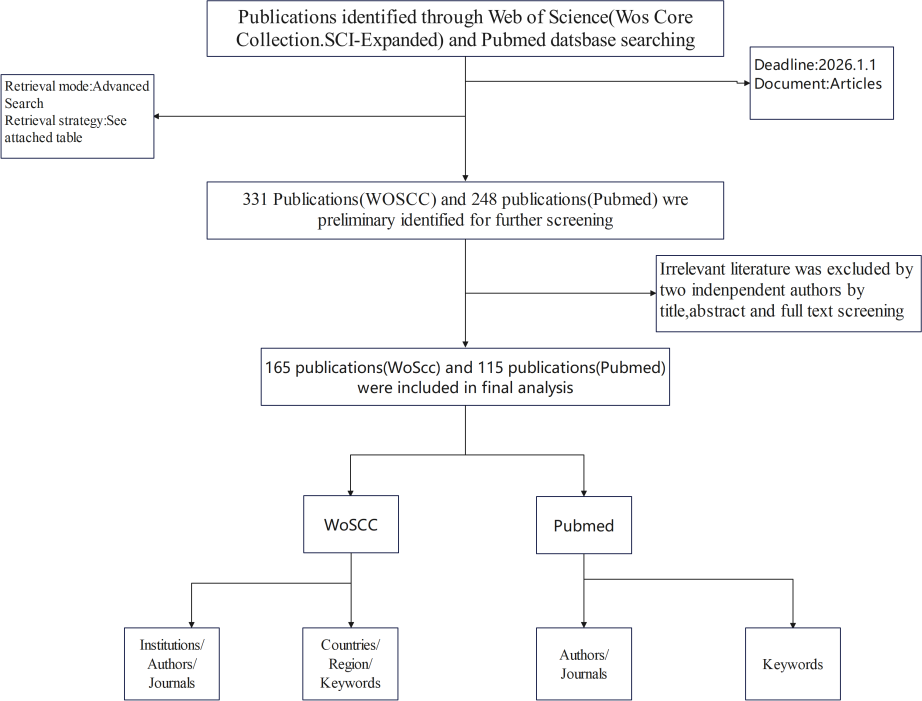


###### **supplementary figure** 1：Retrieval process flowchart for the research


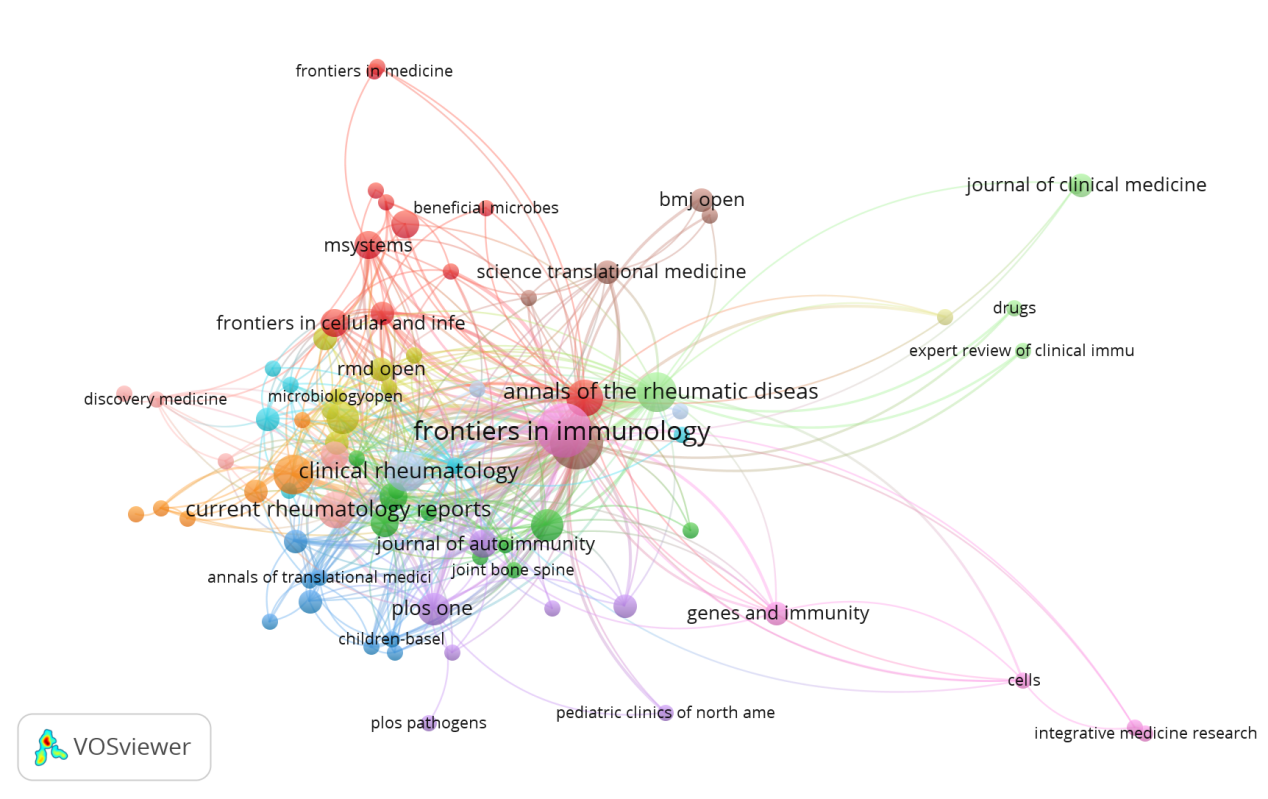


###### **supplementary figure** 2：Network visualization map of journal co-citation analysis generated by VOSviewer.


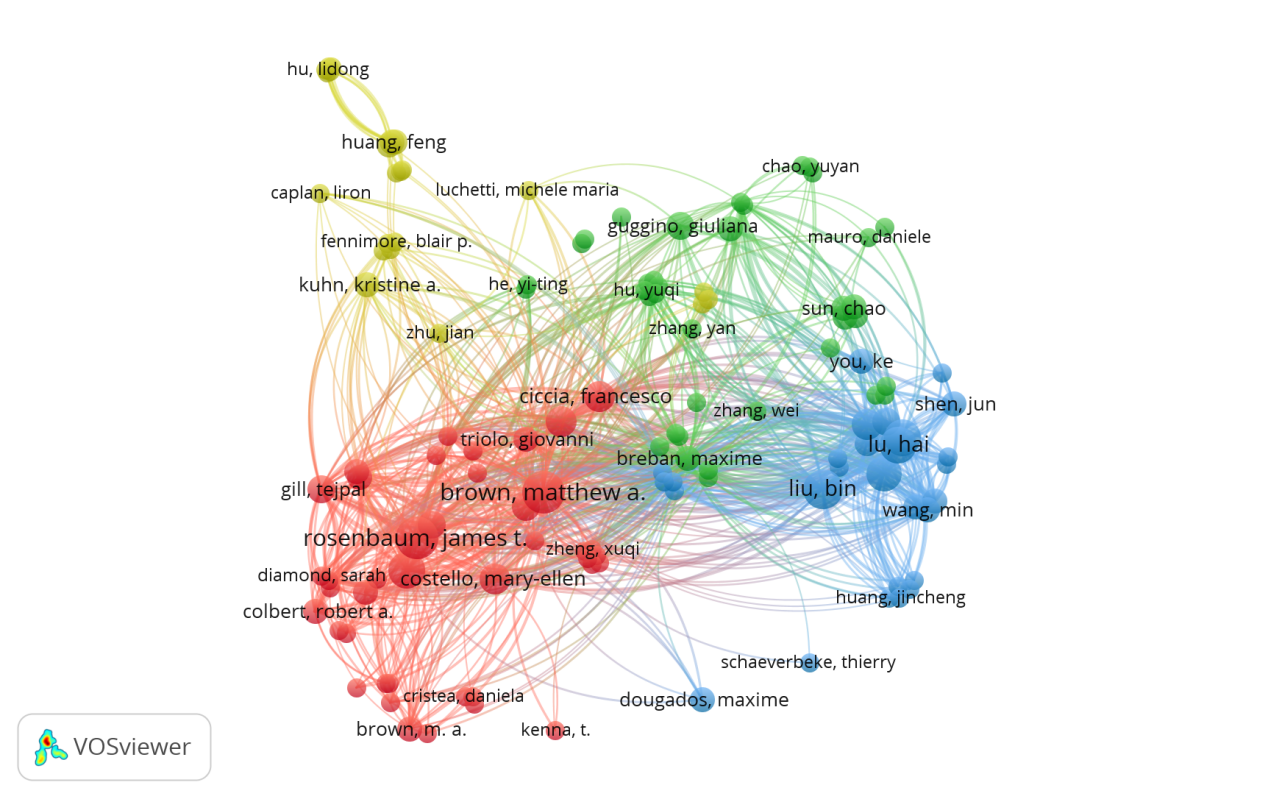


###### **supplementary figure** 3：Network visualization map of author co-citation analysis generated by VOSviewer.

**
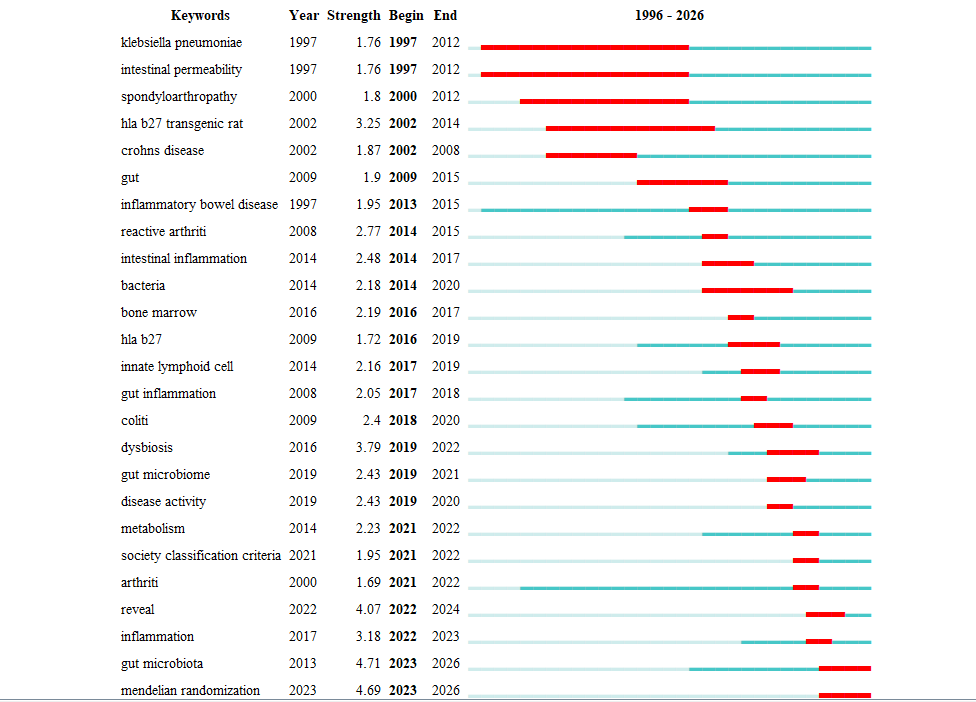
**

**supplementary figure ４** Keywords outbreak

Note:The red color at the end indicates that this keyword is the main research direction for the future

**supplementary Table 1：Top 10 most productive journals.**

| Ranking | Journal | Output | % of 165 | IF  2024 | JCR 2024 |
| --- | --- | --- | --- | --- | --- |
| 1 | \| FRONTIERS IN IMMUNOLOGY \| \| --- \| | 11 | 6.587 | 5.9 | Q1 |
| 2 | ARTHRITIS RHEUMATOLOGY | 10 | 5.988 | 10.9 | Q1 |
| 3 | ANNALS OF THE RHEUMATIC DISEASES | 6 | 3.593 | 20.6 | Q1 |
| 4 | CLINICAL RHEUMATOLOGY | 6 | 3.593 | 2.8 | Q2 |
| 5 | SCIENTIFIC REPORTS | 6 | 3.593 | 3.9 | Q1 |
| 6 | ARTHRITIS RESEARCH THERAPY | 5 | 2.994 | 4.6 | Q1 |
| 7 | CURRENT RHEUMATOLOGY REPORTS | 5 | 2.994 | 3.9 | Q2 |
| 8 | BEST PRACTICE RESEARCH IN CLINICAL RHEUMATOLOGY | 4 | 2.395 | 4.8 | Q1 |
| 9 | INTERNATIONAL JOURNAL OF RHEUMATIC DISEASES | 4 | 2.395 | 2.0 | Q3 |
| 10 | PLOS ONE | 4 | 2.395 | 2.8 | Q2 |

**supplementary Table 2：Top 10 highly cited literature.**

| Ranking | Title | First author | Year | Citations | Citations/Year |
| --- | --- | --- | --- | --- | --- |
| 1 | Intestinal Dysbiosis in Ankylosing Spondylitis | [Costello ME](https://webofscience.clarivate.cn/wos/author/record/6496904) | 2015 | [326](https://webofscience.clarivate.cn/wos/woscc/citing-summary/21394332?type=refid) | 27.17 |
| 2 | [Quantitative metagenomics reveals unique gut microbiome biomarkers in ankylosing spondylitis](https://webofscience.clarivate.cn/wos/woscc/full-record/WOS:000406394700003) | [Wen CP](https://webofscience.clarivate.cn/wos/author/record/55127373) | 2017 | 282 | 28.20 |
| 3 | Faecal microbiota study reveals specific dysbiosis in spondyloarthritis | [Breban M](https://webofscience.clarivate.cn/wos/author/record/1409721) | 2017 | 281 | 28.10 |
| 4 | Dysbiosis and zonulin upregulation alter gut epithelial and vascular barriers in patients with ankylosing spondylitis | Ciccia F | 2017 | 264 | 26.40 |
| 5 | IgA-coated E. coli enriched in Crohn's disease spondyloarthritis promote TH17-dependent inflammation | Viladomiu M | 2017 | 245 | 24.50 |
| 6 | Dialister as a Microbial Marker of Disease Activity in Spondyloarthritis | Tito RY | 2017 | 221 | 22.10 |
| 7 | HLA-B27 and Human β2-Microglobulin Affect the Gut Microbiota of Transgenic Rats | Lin P | 2014 | 198 | 15.23 |
| 8 | Altered microbiota associated with abnormal humoral immune responses to commensal organisms in enthesitis-related arthritis | Stoll ML | 2014 | 159 | 12.23 |
| 9 | HLA Alleles Associated With Risk of Ankylosing Spondylitis and Rheumatoid Arthritis Influence the Gut Microbiome | [Asquith M](https://webofscience.clarivate.cn/wos/author/record/19590943) | 2019 | 143 | 17.88 |
| 10 | Metagenomic profiling of the pro-inflammatory gut microbiota in ankylosing spondylitis | Zhou C | 2020 | 134 | 19.14 |
