# Supplementary material for: Ankylosing spondylitis and the gut microbiome: future research hotspots and trends
Source: Front Immunol. 2026 May 5;17:1784757. doi: 10.3389/fimmu.2026.1784757 (PMC13183638; doi:10.3389/fimmu.2026.1784757)
Supplement: Supplementary file 2 [file Table2.docx]

**Ankylosing spondylitis and the gut microbiome: Future research hotspots and trends.**

**Appendix 1**

**Ankylosing spondylitis and artificial intelligence term**

( Bechterew's Disease or Bechterews Disease or Marie-Struempell Disease or Marie Struempell Disease or Spondylarthritis Ankylopoietica or Rheumatoid Spondylitis or Spondylitis, Rheumatoid or Ankylosing Spondylitis or Ankylosing Spondylarthritis or Ankylosing Spondylarthritides or Spondylarthritides, Ankylosing or Spondylarthritis, Ankylosing or Ankylosing Spondyloarthritis or Ankylosing Spondyloarthritides or Spondyloarthritides, Ankylosing or Spondyloarthritis, Ankylosing or Spondylitis Ankylopoietica or Bechterew Disease or Spondyloarthritis Ankylopoietica ) and ( Gastrointestinal Microbiome OR Gastrointestinal Microbiomes OR Microbiome, Gastrointestinal OR Gut Microbiome OR Gut Microbiomes OR Microbiome, Gut OR Gut Microflora OR Microflora, Gut OR Gut Microbiota OR GutM Microbiotas OR Microbiota, Gut OR Gastrointestinal Flora OR Flora, Gastrointestinal OR Gut Flora OR Flora, Gut OR Gastrointestinal Microbiota OR Gastrointestinal Microbiotas OR Microbiota, Gastrointestinal OR Gastrointestinal Microbial Community OR Gastrointestinal Microbial Communities OR Microbial Community, Gastrointestinal OR Gastrointestinal Microflora OR Microflora, Gastrointestinal OR Gastric Microbiome OR Gastric Microbiomes OR Microbiome, Gastric OR Intestinal Microbiome OR Intestinal Microbiomes OR Microbiome, Intestinal OR Intestinal Microbiota OR Intestinal Microbiotas OR Microbiota, Intestinal OR Intestinal Microflora OR Microflora, Intestinal OR Intestinal Flora OR Flora, Intestinal OR Enteric Bacteria OR Bacteria, Enteric )
